# The Interrelationships between Lactose Intolerance and the Modern Dairy Industry: Global Perspectives in Evolutional and Historical Backgrounds

**DOI:** 10.3390/nu7095340

**Published:** 2015-08-31

**Authors:** Nissim Silanikove, Gabriel Leitner, Uzi Merin

**Affiliations:** 1 Biology of Lactation Laboratory, Institute of Animal Science, Agricultural Research Organization, The Volcani Center, P.O. Box 6, Bet Dagan 50250, Israel; 2 National Mastitis Reference Center, Kimron Veterinary Institute, P.O. Box 12, Bet Dagan 50250, Israel; E-Mail: leitnerg1@gmail.com; 3 Department of Food Quality and Safety, Agricultural Research Organization, The Volcani Center, P.O. Box 6, Bet Dagan 50250, Israel; E-Mail: uzmerin@gmail.com

**Keywords:** lactose intolerance, milk, dairy products, calcium

## Abstract

Humans learned to exploit ruminants as a source of milk about 10,000 years ago. Since then, the use of domesticated ruminants as a source of milk and dairy products has expanded until today when the dairy industry has become one of the largest sectors in the modern food industry, including the spread at the present time to countries such as China and Japan. This review analyzes the reasons for this expansion and flourishing. As reviewed in detail, milk has numerous nutritional advantages, most important being almost an irreplaceable source of dietary calcium, hence justifying the effort required to increase its consumption. On the other hand, widespread lactose intolerance among the adult population is a considerable drawback to dairy-based foods consumption. Over the centuries, three factors allowed humans to overcome limitations imposed by lactose intolerance: (i) mutations, which occurred in particular populations, most notably in the north European Celtic societies and African nomads, in which carriers of the lactose intolerance gene converted from being lactose intolerant to lactose tolerant; (ii) the ability to develop low-lactose products such as cheese and yogurt; and (iii) colon microbiome adaptation, which allow lactose intolerant individuals to overcome its intolerance. However, in a few examples in the last decade, modern dairy products, such as the popular and widespread bio-cultured yogurts, were suspected to be unsuitable for lactose intolerant peoples. In addition, the use of lactose and milk-derived products containing lactose in non-dairy products has become widespread. For these reasons, it is concluded that it might be important and helpful to label food that may contain lactose because such information will allow lactose intolerant groups to control lactose intake within the physiological limitations of ~12 g per a single meal.

## 1. Introduction

The existence of mammary glands is the primary distinguishing characteristic of *Mammalia*, the class of which humans belong to. The term *Mammalia* coined by Carl Linnaeus in 1758 is derived from the Latin word *mamma* (which means mother). All female mammals nurse their young with milk, which is secreted from special glands—the mammary glands. Lactose is the principal carbohydrate in all mammals’ milk, including farm animals that produce milk (Table 1). Human milk contains ~7% lactose, whereas domestic ruminant’s milk, which provides a major part of human nutrition, contains ~5% lactose. Lactose is the most important energy source during the first year of human life, providing almost half of the total energy required by infants [1]. Lactose is a disaccharide that is formed within the Golgi apparatus of the epithelial cells of mammary glands by condensation of glucose and galactose (galactose derived from the Greek word *γάλακτ*–galakt, which means milk) by the enzyme lactose synthase [2]. Unlike other mono-saccharides, such as glucose and fructose, the dietary source of galactose from food is scarce and therefore the vast majority of galactose is metabolized from glucose and glycerol in the mammary gland epithelial cells through hexoneogenesis [3]. Lactose is the major osmotically-active molecule which is impermeable through membranes and is stored for secretion in secretory vesicles. The high concentration of lactose in secretory vesicles accounts for their osmotic swelling and dictates the volume of milk secretion [4].

**Table 1:** Fat, protein, and lactose content in milk of different mammalian species [5].

Specie	Fat	Protein	Lactose
	g/L		
Human	11	42	70
Cow	35–45	30–36	47–50
Sheep	60–80	50–65	44–48
Goat	30–34	27–37	42–48
Buffalo	70–74	38–44	48–50

Since any disaccharide such as sucrose and dextrose (composed of monosaccharides commonly available from food—glucose and fructose) can function as an osmotic driver of milk secretion, it could be assumed that the evolvement of lactose as the main carbohydrate in milk or the presence of galactose in the infant diet has particular evolutionary advantages. Galactose and its derivatives play a central role in higher eukaryotes in the biosynthesis of complex carbohydrates, glycoproteins, and glycolipids [6]. Galactooligosaccharides have prebiotic properties and their ingestion promotes positive effects on gut microflora and health [7,8]. Galactose, through its conversion to *N*-acetylgalactosamine is one of the six carbohydrates used for construction of gangliosides, which are essential membrane components that play an important role in signal transduction and immunity [9]. Thus, while data supports the concept that lactose plays an essential role in infant nutrition, research in this field lags considerably behind its importance.

In order to digest and absorb lactose from the digestive system it is hydrolyzed in the small intestines of mammal’s infants into β-d-glucose and β-d-galactose by the enzyme lactase (β-d-galactosidase; β-d-galactoside galactohydrolase, EC 3.2.1.23), secreted by the villi of epithelial cells [10].

Most humans normally cease to produce lactase after weaning and as a result become lactose intolerant. It is, therefore, not surprising that as adults, as much as 75% of the world’s human population is intolerant to ingested dietary lactose [11,12,13,14,15,16,17,18,19,20,21]. Some populations, however, have developed lactose persistence, in which lactase production continues into adulthood [22,23,24,25,26,27]. The importance of these phenomena for the development of the dairy industry will be discussed below.

## 2. Interrelationships among Civilization, Dairy Technology, Recent Mutation of the Lactase Gene, and the Development of the Dairy Industry

Humans first learned to exploit ruminants following their domestication during the Neolithic Revolution, also known as the Agricultural Revolution (Table 2). Cattle, sheep, and goats were first domesticated in Mesopotamia (the area of the Tigris–Euphrates river system in Asia), commonly considered the cradle of modern civilization in Western culture. Water buffalo and yak were domesticated somewhat later in China, India, and Tibet (Table 2). Until recently, the theory of the archeologist Andrew Sherratt that animals were initially kept for meat was widely accepted. Exploitation of domestic animals for dairy, hair, and labor began much later in a separate revolution in the 4th millennium BC [28,29]. However, Sherratt’s model is not supported by recent findings. Animal skins and inflated internal organs, particularly the rumen, have provided storage vessels for a range of foodstuffs since ancient times. The process of cheese-making was probably discovered accidentally by storing milk in a container made from the stomach of a ruminant, resulting in the peculiar phenomena in which the milk turned into curd and whey by the residual rennin enzyme in the stomach [30]. Based on the analysis of lipid residues in prehistoric pottery, it was concluded that dairying was practiced in the early phases of agriculture in Southwest Asia, as early as the 7th millennium BC [31,32].

**Table 2:** Approximate dates and locations of ruminant domestication.

Species	Time	Location	Reference
Sheep (*Ovis orientalis aries*)	between 11,000 BCE and 9000 BCE	Mesopotamia	[33]
Goat (*Capra aegagrus hircus*)	8000 BCE	Mesopotamia	[34]
Cow (*Bos primigenius taurus*)	10,800–10,200 BCE	Mesopotamia	[35]
Water buffalo (Bubalus bubalis)	5000–7000 BCE	India, China	[36,37]
Yak (Bos grunniens)	4500 BCE	Tibet	[38]

Prehistoric evidence indicates that dairying evolved in different ways in different areas, in part due to different environmental conditions and in part due to different cultural choices of early farmers [30,31,32]. Furthermore, the evidence suggests that at least in some Neolithic societies, even before 6500 BC, humans learned to process milk into cheese. Evidence for the spread of cheese-making in early human societies came from the Sumerian cuneiform texts of Third Dynasty of Ur (~2000 BC), Egyptian tomb murals from the same period and Late Bronze Age Minoan-Mycenaean in Crete. By the Roman times, cheese had become a daily basic food and a staple of long-distance commerce [29,30]. Most of the prevailing cheeses in Western societies were developed during the Middle-Ages and today ~400 types of cheeses under ~1000 names are produced in England, France, and Italy [39]. It is interesting to note that cheese, which is naturally low in lactose, developed mostly in areas such as the Middle East and Southern Europe, where lactose intolerance was widespread. Technological advances have made it possible to store milk for a long time by converting it to products with extended shelf-life, enabling the utilization of milk in spite of the prevalence of lactose intolerance in the population.

Another means enabling the spread of dairying in certain populations were genetic niche constructions, derived from a mutation in the lactase gene enabling the digestion of lactose by adult humans [40]. Modern descendants of hunter-gatherer populations, their ancestors (e.g., Native Americans) and most human populations before the Neolithic Revolution were mostly lactose intolerant [41]. Genetic studies suggest that the oldest mutations associated with lactase persistence reached appreciable levels in human populations only in the last ten thousand years, which coincide with the Neolithic Revolution and dairy animal domestication [42]. Therefore, lactase persistence is often cited as an example of recent human evolution [40,41,42]. Due to the fact that lactase persistence is a genetic trait, closely associated with animal husbandry-cultural traits, it is considered as gene-culture co-evolution (or niche construction) in the mutual human-animal symbiosis, occurring simultaneously with the advent of agriculture [40]. Theories on why the ability to digest lactose might be advantageous are based on nutritional benefits: milk as a source of energy and water in times of drought and increased calcium absorption, helping the prevention of rickets and osteomalacia in low-sunlight regions [42]. In the Northern European populations, the spread of the lactase persistence allele is correlated most closely with positive selection of dense bone buildup due to added vitamin D to the diet which promotes calcium absorption [41], while in African populations, where vitamin D deficiency is not as much of an issue, the spread of the allele most closely correlates with added calories and nutrition from pastoralism [43,44]. Records from the Roman period indicate that the people of Northern Europe, particularly Britain and Germany, drank unprocessed milk. This corresponds very closely to modern European distribution of lactose intolerance, where the people of Britain, Germany, and Scandinavia possess high tolerance and those of Southern Europe, especially Italy, have lower tolerance. In our time, there is no clear trend between per capita milk consumption and lactose tolerance (Table 3 *vs.* Table 4), or between cheese intake and lactose tolerance (Table 3 *vs.* Table 5). Thus, milk and cheese consumption in different geographical regions seems to depend on the wealth of the population and cultural fashions.

**Table 3:** Lactose intolerance in different human groups.

Human Group	Individuals Examined	Intolerance (%)	Reference
Dutch	N/A	1	[27]
Europeans in Australia	160	4	[13,18]
British	N/A	5–15	[26]
Central Italians	65	19	[13,16]
Indians	N/A	20	[11,12]
Australian Aborigines	44	85	[11,12]
African Bantu	59	89	[11,12]
Asian Americans	N/A	90	[11,12]
Chinese	71	95	[17]
Southeast Asians	N/A	98	[20]
Thais	134	98	[11,12]
Native Americans	24	100	[11,12]

**Table 4:** Per capita consumption of milk (L) and milk products (kg) in various countries–2011 [45].

Country	Fluid Milk	Cheeses	Butter
**North America**			
USA	75.8	15.1	2.8
Canada	78.4	12.3	2.5
**Europe (EU26)**	62.8	17.1	3.6
Ireland	135.6	6.7	2.4
Finland	127.0	22.5	4.1
UK	105.9	10.9	3.0
Sweden	90.1	19.1	1.7
France	55.5	26.3	7.5
Italy	54.2	21.8	2.3
Germany	51.8	22.9	5.9
Greece	49.1	23.4	0.7
Netherlands	47.5	19.4	3.3
**Other**			
Australia	105.3	11.7	4.0
Brazil	55.7	3.6	0.4
India	39.5	-	3.5
China	9.1	-	0.1

**Table 5:** Milk and dairy products consumption in the USA (except cheese) has steadily declined since the mid-1980s. (USDA, Economic Research Service, Food Availability (Per Capita) Data System).

	1970	1980	1990	2000	2012
	*Per capita availability (pounds)*				
Fluid milk and cream	273.8	245.0	232.8	209.9	198.8
Butter	5.4	4.5	4.4	4.5	5.6
Cheese and cottage cheese	16.4	21.9	27.9	32.5	36.0
Frozen dairy products	25.8	23.9	26.1	28.1	23.9
Evaporated and condensed milk and dry dairy products	17.7	10.5	11.6	9.0	11.6
Total	339.2	305.8	302.8	283.9	275.9

An additional major mechanism for coping with lactose intake is through adaptation, principally through microbiome colonic adaptation. A review which covers adaptation to lactose intake and the prebiotic properties of lactose is available to the readers in this special issue [46]; therefore, these aspects are not covered here in detail. It may be concluded that, in addition to habitual learning to consume milk within physiological limits, both technological development and genetic evolution contributed to the widespread of dairy industry around the globe.

## 3. A Short Overview on the Relative Size of the Global Dairy Industry

Milk is a bulky liquid susceptible to spoilage by microorganisms that shortens its shelf-life. Most countries produce their own milk and milk products locally. New Zealand and Australia are exceptions that produce surplus amounts for the international market. However, the structure of the dairy industry varies in different parts of the world, from many small family dairies to a few large industrial dairies.

The share of total dietary energy intake coming from dairy products is ~14% in developed countries and is quite constant. In contrast, in developing counties energy intake coming from dairy products is only ~4%, but is continually increasing [47,48]. Recently, there is a trend towards reduction in milk consumption in the USA and some Western countries, despite the prevalence of a relatively high proportion of a lactose-persistent population. However, the reduction in milk consumption is, to a large extent, balanced by increased consumption of fermented products (yogurts) and cheese, as such or as additives to products such as pizza and cakes (Table 5).

In developing countries, the production growth of fermented dairy products has significantly outpaced that of developed countries, particularly in countries with large populations and with increases of income such as China, India, and Turkey (Table 3) [47]. Cow milk dominates global milk production, but milk from other animals is important in specific areas, particularly for family-type production systems in developing regions. Globally, cow milk represents 85% of the world production and at least 80% of the total production in all regions, except South Asia, where its share is less than half (44%) [47]. Geographically, Asia has turned into the largest milk producer in 2013 with a 13 year average increase of 5.2% with the EU and North and Central America losing their dominancy (Table 6) [49]. In addition to cow’s milk, only buffalo milk makes a substantial contribution on the global level (as for 2013), accounting for 13.2% of worldwide total milk production (about 23% in developing countries), while the contribution of milk from goats (2.4%) and sheep (1.3%) is much lower [47,49]. However, the low proportion of the contribution of the dairy goat sector to total milk production is misleading: About 80% of the goats around the world are located in tropical areas of Asia, Africa, and South America. The number of cows in those areas is low compared to goats and because of high population density, the total of milk and other goat dairy products consumed in these parts of the world are by far greater than that of other dairy animals [50].

**Table 6:** World cow’s milk production 2000–2013 (1000 tonnes) [49].

Region	2000	2005	2010	2012	2013	Annual Growth '12-'13 (%)	CAGR * '00-'13 (%)
Asia	94,884	131,350	165,990	180,051	183,504	+1.9	+5.2
EU 28	150,071	150,448	149,810	152,714	154,041	+0.9	+0.2
North & Central America (**)	97,963	103,005	112,089	116,169	116,599	+0.4	01.3
South America	47,466	53,461	64,868	68,820	70,096	+1.9	+3.0
Other Europe	58,405	60,378	59,406	59,274	58,021	-2.1	-0.1
Africa	19,272	26,637	31,765	32,982	34,089	+3.4	+4.5
Oceania	24,260	25,621	26,612	30,120	29,788	-1.1	+1.6
**World**	**492,321**	**550,900**	**610,539**	**640,130**	**646,138**	**+0.9**	**+2.1**

* Compound annual growth rate; ** Including Caribbean.

## 4. How Do Humans Cope with Lactose Intolerance?

Lactose intolerance is a syndrome of diarrhea, abdominal pain, flatulence, and/or bloating that occur after ingestion of lactose. The symptoms of lactose intolerance result from secretion of catabolic fragments of bacterial fermentation of undigested lactose in the colon**.** Four types of lactase deficiency may lead to lactose intolerance (Box 1.) [51].

Box 1 **Types of lactase deficiency which may lead to lactose intolerance.**

- Primary lactase deficiency, also called lactase non-persistence, is the most common type of lactase deficiency. In people with this condition, lactase production declines over time. This decline often begins at about age 2; however, it may begin later. Children who have lactase deficiency may not experience symptoms of lactose intolerance until late adolescence or adulthood.
- Secondary lactase deficiency results from injury to the small intestine. Infection, diseases, or other problems may injure the small intestine. Treating the underlying cause usually improves the lactose tolerance.
- Developmental lactase deficiency may occur in infants born prematurely. This condition usually lasts for only a short time after they are born.
- Congenital lactase deficiency is an extremely rare disorder in which the small intestine produces little or no lactase enzyme from birth. Genes inherited from parents cause this disorder.

Lactose intolerance may be troublesome but is not considered a condition requiring medical treatment in societies where the diet contains relatively few dairy products. Dairy products currently available on the market provide solutions to the problem. The main concern of leading health institutions, such as the American National Institute of Health (NIH), is that lactose intolerance may affect people’s health if it keeps them from consuming enough essential nutrients, such as calcium and vitamin D. Calcium is essential at all ages for the growth and maintenance of bones. Shortage of calcium intake in children and adults may lead to less dense bones that can easily fracture later in life, a condition called osteoporosis [51].

Since most people with lactose intolerance can tolerate some amount of lactose in their diet they do not need to avoid milk or milk products completely. There are considerable variations in the amount of lactose that can be tolerated among lactose intolerant individuals. NIH experts suggest that adults and adolescents with lactose mal-absorption could eat or drink at least 12 g of lactose (the amount of lactose in 1 cup of milk) without symptoms or with only minor symptoms. Increasing lactose consumption may be possible if taken with meals or in small amounts throughout the day [51,52]. Although no way to reinstate lactase production has been found as of 2015, some individuals have reported that their intolerance varies over time, depending on health status, pregnancy and adaptation to lactose intake [53], particularly through adaptation of the colon microbial system to lactose [54].

The main dairy products on the market and their relationship to lactose intolerance are described in the following sections.

### 4.1. Milk and Milk Products

A gradual introduction of small amounts of milk or milk products may help some people adapt to lactose with minor symptoms [55]. Often, people can better tolerate milk or milk products by having them with meals, such as having milk with cereal or having cheese with crackers. People with lactose intolerance are generally more likely to tolerate hard cheeses, such as Cheddar or Swiss, than a glass of milk. A 43 g serving of mature-hard cheese contains less than 1 g of lactose, while a single cup serving of milk contains about 11 to 13 g of lactose (Table 7). Increasing the intake of lactose above ~12 g/day will likely cause more and more intense symptoms in a dose-response manner [55].

*Lactose-free and lactose-reduced milk and milk products:* Lactose-free and lactose-reduced milk and milk products are available at most supermarkets in Western countries and are identical, nutritionally, to regular milk and milk products. Manufacturers treat milk with the enzyme lactase to make it almost lactose-free, without changing its shelf-life. This enzyme breaks down the lactose in the milk into two digestible monosaccharides, *i.e.*, glucose and galactose, thus attaining a slightly sweeter taste than regular milk [56,57].

*Lactase products:* For people who are lactose intolerant, it is possible to use lactase tablets and drops to improve digestion of dairy products. The lactase enzyme in the tablets digests the lactose in the food, thus reducing the chances of developing digestive symptoms. However, it is important to check with a health care provider before using these products because some groups, such as young children and pregnant and breastfeeding women, should avoid its use because of lack of sufficient safety information and fear of possible allergic responses [58].

**Table 7:** Daily requirements of calcium by age and comparative serving equivalents of common dairy sources.

		Milk (/100 mL)	Plain Yogurt (/100 mL)	Common Cheeses (Cheddar, Provolone, Mozzarella, *etc.*) (/serving—44 g)
Energy (kcal)		102	148	93
Lactose (g)		5	2.5–3 (3.8–5.0 in bio-yogurts *)	0.3–1
Calcium (mg)		105	132	301
Calcium/lactose ratio (mg·g^−1^)		21	30–48	301–1003
Age (year)	Calcium Needed	Amount Needed to Provide AI for Calcium		
	(AI;** mg·day^−1^)	cups (220 mL)		g
1–3	500	2.3	1.8	108
4–8	800	3.7	2.9	176
9–18	1300	6.0	4.7	286
19–50	1000	4.6	3.6	220
51+	1200	5.5	4.3	264

* See text for special discussion on bio-yogurts; ** Adequate intake.

*Yogurt and other fermented products*: Yogurt (derived from Turkish: yoğurt) is a sour-flavored thick gel product containing all the milk constituents with reduced lactose content. It is produced by bacterial fermentation of the milk lactose into lactic acid, which reduces the milk pH. Traditionally, dairy yogurt is produced using a bacteria culture of *Lactobacillus delbrueckii subsp. bulgaricus* and *Streptococcus thermophilus*. This yogurt is nutritionally rich in protein, calcium, vitamin D, riboflavin, vitamin B6, and B12 [59]. Yogurts are also rich in probiotics (live microorganisms which improve the health status of the host by exerting beneficial effects in the gastrointestinal tract) and prebiotic (undigested compounds formed during fermentation that allows specific changes, both in the composition and/or activity in the gastrointestinal microflora that confers benefits upon host well-being and health) components [60,61].

Lactose-intolerant individuals may tolerate yogurt better than milk due to the partial conversion of the lactose to glucose and galactose by the bacterial fermentation. In addition, it contains the enzyme lactase produced by the bacteria cultures used to make the yogurt which may help to digest lactose in the intestine [62,63]. Many additional varieties of fermented products other than yogurt are produced from milk in different parts of the world such as kefir, sour cream, leben, labaneh, mursik, and viili to mention a few. From the point of view of lactose intolerance, these products most likely have advantages similar to yogurt, including reduced lactose content and increased lactase content.

*Modern forms of cultured bio-yogurts*: The manufacture of cultured dairy products represents the second most important fermentation industry (after the production of alcoholic drinks). Cultured bio-yogurts, or cultured milk (also known as pro-biotic yogurts) and pro-biotic drinking yogurt were the fastest growing dairy product sector between 1998 and 2003 [64]. More than 50% of the world yogurt market in Western countries nowadays is dominated by several international manufacturers, which produce cultured bio-yogurts, or cultured milk blends [65]. Bio-yogurt and cultured milk blends contain additional bacterial strains—Bifidobacteria and *Lactobacillus acidophilus*, which supposedly improve its pre-biotic and pro-biotic properties, thus enhancing its health benefits. A survey of leading local and international brands sold in Israel showed that the lactose content in five brands utilizing the classical cultures, ranged between 2.52% to 2.93%. In contrast, the lactose content in six brands of bio-yogurts ranged between 3.75% and 5.05% [66]. In comparison, it was found that the galactose content in the classical yogurt ranged between 2.60% and 2.93%, whereas in the bio-yogurts it ranged between 1.62% and 2.18%. The only exceptional product was Actimel, a probiotic yogurt-type drink produced by the French company Danone since 1993, in which lactose content was 3.09% and galactose content was 2.66%, closer to that found in products termed yogurts. The data from the Israeli survey strongly suggest that most bio-yogurts on the Israeli market are not suitable for lactose-intolerant people. In addition, since these products are produced by licensed international brands, this conclusion might have wider implications. In one of the products the sum of lactose and galactose content exceeded 7%, strongly suggesting that the producer used lactose as a carbohydrate additive [66]. We suggest legally requiring dairy producers to indicate the lactose content in their products, thus enabling people with lactose intolerance to control their daily lactose intake more easily.

*Cheese*: As mentioned above, cheese is probably the oldest dairy product. The first step in cheese-making is separating the milk into a very moist gel, known as curd and milk serum, *i.e.*, whey. The main protein in milk, casein, is packed in the form of micelle. Caseins in milk of cows, goats, and sheep are composed of five sub-types, α-, β-, αs_1_-, αs_2_-, and κ-casein. κ-casein is responsible for preventing the casein micelles from sticking to each other by ionic charge repulsion. Acidifying the milk or treating it with a coagulating enzyme, such as rennin (an enzymatic complex produced in stomachs of ruminant mammals) or a combination of both treatments, remove the protecting effect of κ-casein from the micelles and cause its collision to form a fine coagulum that entraps the milk fat globules. After disturbing the coagulum by cutting it, over 80% of the milk volume is expelled as whey and a curd is formed. The curd is composed of casein along with calcium and other divalent mineral salts, which are firmly associated with casein and milk fat. The whey is composed of water, lactose and truly soluble proteins such as α-lactalbumin, β-lactoglobulin, bovine serum albumin, along with some enzymes and protein fractions (around one third of the total proteins in milk) and monovalent ions, mostly K and Na [67]. Numerous varieties of cheeses with different taste, aroma and texture are produced around the world using this general scheme [68]. Due to the fact that the separation of curd from whey is not complete, hard and fresh cheeses contain some lactose (Table 7). A portion (43 g) of fresh cheese such as cottage cheese contains lactose well within the recommended tolerance limit of 12 g per day. The content of lactose in hard-matured (long-ripened) cheeses can be very low and thus can be tolerated by most people suffering from congenital lactose intolerance and galactosemia [69]. Since precaution is a must for such conditions, it is suggested that producers provide information on the lactose content in cheeses, particularly in fresh cheeses, as well as for many modern brands of commercial dairy foods to which milk solids are added. Such information can provide the consumer with a clearer idea regarding the suitability of a particular product for his dietary needs.

*Caseinates*: Caseinates are the soluble salts of acid casein (the main protein in milk) that are produced by the dairy industry as dietary supplements and food ingredients. Caseinates serve as the main source of protein in most bottle-fed infant formulas. Caseinates are virtually lactose free.

*Whey protein isolate (or whey isolate)*: Whey proteins are isolated by dairy producers from whey to serve as dietary supplement and food ingredient [70].Whey can be processed to yield whey protein in three forms: whey isolate, whey concentrate, or whey hydrolysate. The main difference between the three products is the percent of protein content and level of hydrolysis. Whey isolates contain the higher percentage of pure protein and can be pure enough to be virtually lactose free.

*Butter*: During the butter-making process, the majority of the milk water soluble components are separated from the fatty matter. Lactose, being a water-soluble molecule, is largely expelled in the buttermilk, but some lactose remains in small quantities in the butter, unless it is also fermented to produce cultured butter. Clarified butter, however, contains very little lactose and is safe for most lactose-intolerant people.

*Lactose in nondairy products*: Lactose is a common commercial food additive [71], regularly used by the food industry due to its low price, its texture, flavor, and adhesive qualities. Lactose is found in foods, such as processed meats (sausages/hot dogs, sliced meats, pâtés), gravy stock powder, margarines, sliced bread, breakfast cereals, potato chips, processed foods, prepared meals, meal replacements (powders and bars), protein supplements (powders and bars), and even in beer of the milk stout style and medications (especially pills). Some barbecue sauces and liquid cheeses used in fast-food restaurants may also contain lactose. Labels such as lactoserum (French—whey), whey, milk solids, modified milk ingredients *etc.*, indicate that these products probably contain lactose. Kosher products labeled as Pareve (Yiddish—with no meat or dairy origin) or Fleishig (German—meat origin) are free of milk and, thus, of lactose. However, if a “D” (for “dairy”) is present next to the circled “K”, “U”, or other kosher “qualifications”, the food product may contain milk solids or may simply indicate the product was produced on equipment shared with other products containing milk derivatives. From the point view of lactose intolerance, it would be helpful to indicate the content of lactose in nondairy products in order to decrease the risk of consuming above 12 g lactose per meal.

### 4.2. Integrative Discussion

As discussed above, two main factors contributed to the widespread use of milk and dairy products in the diet in different geographical areas and different cultures around the globe. One is the genetic change that resulted in the ability of humans to digest lactose after weaning and the second is the development of products such as cheese and fermented milk products that allow storing, trade and digesting these products by lactose intolerant humans. Due to the prevalence of lactose intolerance, we believe that the technological development of hundreds of varieties of cheeses and fermented products in different parts of the world enabled the dairy industry to develop into one the most important and diverse sectors in the food industry. The main reason for the development of this large diversity is that milk and dairy products provide important, almost irreplaceable, nutritional advantages, which proved themselves in different cultures over the course of 10,000 years of human development.

**Figure 1:**
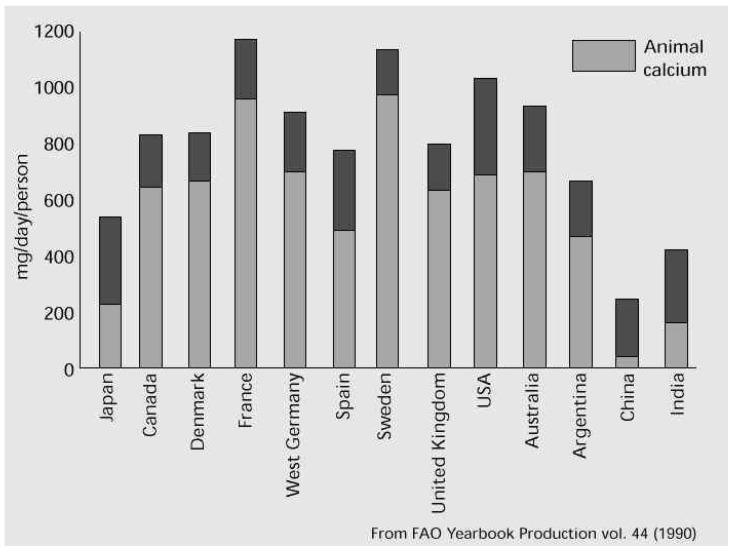
Calcium intake in different countries (animal calcium is provided mainly by dairy products). Note: this information does not include recent changes in dairy consumption in East Asian countries. The calcium intake, due to lack of milk intake, in many African countries is very low in comparison to requirements (see text).

Milk and dairy products are categorized as nutrient-dense foods, *i.e.*, foods that deliver many nutrients and are relevant to health throughout the life cycle (Table 8) [50]. Milk has many additional nutritional advantages, and those related to the presence of lactose are topic of other reviews in this special issue. However, as already outlined, milk and dairy products have one advantage over all other foods as a source of calcium, providing daily nutritional requirements, which was most likely one of the most important drives for the development of the dairy industry. By increasing the proportion of cheese and yogurts, these requirements can be met with reasonable intake of energy (fat) while keeping lactose intake below 12 g per meal. Thus, dairy products can provide calcium needs of lactose intolerant people even to those suffering from galactosemia by maintaining more stringent regulations of dairy products consumed. Some recommendations from the USA and UK on how to meet calcium needs by a combination of milk and dairy products at different ages are presented in Table 7, Table 8, Table 9 and Table 10. In comparison, the calcium content in common vegetarian sources and its contribution to daily needs of a grown-up person is as follows: white beans: 191 mg (19% of the reference nutrient intake—RNI) in one canned cup; Dried Figures: 107 mg (10% RNI) in eight whole dried figs; kale: 188 mg (19% of the daily value—DV) in two raw (chopped) cups; almonds: 72 mg (7% RNI) in a ¼ of dry roasted cup (about 20 nuts); oranges: 65 mg (6% RNI) in one medium fruit; turnip greens: 197 mg (20% RNI) in one cooked (chopped) cup; sesame seeds: 88 mg (9% RNI) in 1 tablespoon; and seaweed: 126 mg (13% RNI) in about 1 cup raw cup [72]. Though it is frequently stated in Internet sources (such as the one cited above) that calcium needs can be met by vegetarian source that could turn out to be a difficult task. When comparing the data regarding contribution to needs in Table 7, Table 8, Table 9 and Table 10, it seems that it would require considerable planning and effort by individuals, what is usually unattainable by the majority of the population. Indeed, from a national nutrition survey carried out during 2001–2002 in the US, it was concluded that adequate intake for calcium by adolescents (9–18 years of age) cannot be met with dairy-free diets while meeting other nutrient recommendations [73]. On the other hand, dietary surveys of the sources of calcium in human diet show that most calcium requirements in developed countries are provided by milk and dairy products, whereas lack of adequate calcium intake in developing countries is related to low intake of dairy products. The data clearly show that calcium intake in most developing countries lags considerably below nutritional needs. In developed countries, 50% to 90% of calcium needs of infant to pubertal boys are provided by milk and dairy products (Figure 1, Table 8). The consequence of shortage in calcium and phosphorus intake might be nutritional rickets, which could be reflected by soft and weakened bones in infants. In African children at 18 months of age, calcium intake of ~200 mg per day with apparent exposure to sunlight was suggested as the cause of delayed motor development, hypotonia, short stature, and knock-knees or bowed legs [70,73,74,75,76,77,78,79]. Even in Western countries, low dietary calcium intake might be responsible for nutritional rickets in toddlers whose dairy intake is limited [80,81]. High prevalence of rickets is particularly evident in those insisting on vegetarian regime, such as the macrobiotic diet (high whole grain diet supplemented with vegetables) [82].

**Table 8:** Percent contribution of dairy products (milk, milk drinks, yogurts, cheeses, and dairy desserts) to key nutrient intakes in children and adolescents in developed countries *.

Nutrient	Percent Contribution
Energy	13–25
Fat	9–24
Calcium	53–73
Phosphorus	29–31
Iodine	35–50
Zinc	16–39
Potassium	21–22
Retinol	24–42
Vitamin B12	23–59
Riboflavin	29–38

*: Based on data compiled in [83].

**Table 9:** Daily recommendation of drinking milk by the United State Department of Agriculture (USDA) [84].

	Age (year)	Serving (Cup)		Age (year)	Serving (Cup)
Children	2–3	2	Women	19–30	3
	4–8	2 ½		31–50	3
Girls	9–13	3		51+	3
	14–18	3	Men	19–30	3
Boys	9–13	3		31–50	3
	14–18	3		51+	3

**Table 10:** Supply of calcium by a combination of milk and dairy products [85].

Age/Sex	RNI* for Calcium (mg/day)	Dairy Portion Sizes
0–12 months	525	No cows’ milk as a drink for babies under 12 months. Breastfeeding is best, followed by cows’ milk formula. Soya-based formula should be used only under medical advice. Cheese and yogurt can be given from 6 months.
1–3 years	350	100 mL whole/semi-skimmed milk **, 80 g yogurt, 15 g cheese. These portion sizes in total provide approximately 360 mg calcium.
4–6 years	450	130 mL semi-skimmed milk, 100 g yogurt, 20 g cheese. These portion sizes in total provide approximately 465 mg calcium.
7–10 years	550	150 mL semi-skimmed milk, 125 g yogurt, 25 g cheese. These portion sizes in total provide approximately 570 mg calcium.
11–18 years, male	1000	250 mL semi-skimmed milk, 200 g pot of yogurt, 45 g low fat cheese. These portion sizes in total provide approximately 1002 mg calcium.
11–18 years, female	800	200 mL semi-skimmed milk, 200 g pot of yogurt, 30 g of low fat cheese (small matchbox size). These portion sizes in total provide approximately 842 mg calcium.
19–50 years	700	200 mL semi-skimmed milk, 150 g pot of low-fat yogurt, 30 g cheese (small matchbox size). These portion sizes in total provide approximately 710 mg calcium.
0+ years	700	200 ml semi-skimmed milk, 150 g pot of low-fat yogurt, 30 g of cheese (small matchbox size). These portion sizes in total provide approximately 710 mg of calcium.
Pregnancy	700	200 ml semi-skimmed milk, 150 g pot of low-fat yogurt, 30 g of cheese (small matchbox size). These portion sizes in total provide approximately 710 mg of calcium.
Lactation	RNI for age group plus another 550 mg increment, *i.e.*, if lactating youngster, then 800 + 550 mg/day, if lactating adult then 700 + 550 mg/day	To achieve the RNI for calcium during lactation, teenage or adult mums will need to consume more than the portion sizes given above.

* RNI—reference nutrient intake. The amount estimated to be sufficient for 97% of a specified population group; ** Semi-skimmed milk may be introduced to children from the age of two if they are good eaters, otherwise whole milk may continue to be given.

## 5. Conclusions

The dairy industry has developed into one of the largest food industry sectors despite the fact that most humans are lactose intolerant. In some human populations, lactose tolerance developed about 10,000 years ago. However, most humans have learned to cope with lactose intolerance by learning to consume milk within their physiological limitations, improving their ability to consume lactose by microbiome adaptation and by producing wide varieties of low-lactose dairy products. Milk and dairy products provide many nutritional advantages, the most important being the consumption of calcium. The recent spread of milk usage in countries, such as China and Japan in which the vast majority of the population is lactose intolerant, is a vivid example of the realization of the population, health care professionals, and public opinion leaders in these countries regarding the importance of milk as a source of essential nutrients. However, a continuing source of concern is that some dairy products produced by large manufacturers as lactose intolerance-friendly products, whereas in reality their servings contain lactose in amounts which are close or even exceed the physiological limitations of about 12 g lactose per meal. Thus, it is recommended that food producers using milk and milk-derived products be required to specify, along with the nutritional information, the content of lactose in their products.
